# Temporal and Spatial Variation of Toxic Metal Concentrations in Cultivated Soil in Jiaxing, Zhejiang Province, China: Characteristics and Mechanisms

**DOI:** 10.3390/toxics12060390

**Published:** 2024-05-26

**Authors:** Mengzhuo Cao, Yanbo Jia, Xin Lu, Jinfa Huang, Yanlai Yao, Leidong Hong, Weijing Zhu, Weiping Wang, Fengxiang Zhu, Chunlai Hong

**Affiliations:** 1 Institute of Environment, Resource, Soil and Fertilizer, Zhejiang Academy of Agricultural Sciences, Hangzhou 310021, China; cmz201309@163.com (M.C.); q2080252919@163.com (X.L.); yaoyl@zaas.an.cn (Y.Y.); hongld@zaas.ac.cn (L.H.); zhuwj@zaas.ac.cn (W.Z.); wangwp@zaas.ac.cn (W.W.); zhufx@zaas.ac.cn (F.Z.); 2 Hangzhou Institution of Food and Drug Control, Hangzhou 310022, China; jiaboshi2002@sina.com; 3 Jiaxing Soil and Fertilizer Plant Protection and Rural Energy Station, Jiaxing Agriculture and Rural Bureau, Jiaxing 314050, China; jxstfz@126.com; 4 Shanghai Huadi Environmental Technology Co., Ltd., Shanghai 201803, China

**Keywords:** agriculture, concentration, safe limits, national risk screening value, pollution

## Abstract

The toxic metal (As, Cd, Cr, Cu, Hg, Ni, Pb, and Zn) pollution in 250 agricultural soil samples representing the urban area of Jiaxing was studied to investigate the temporal and spatial variations. Compared to the early 1990s, the pollution level has increased. Industry and urbanization were the main factors causing toxic metal pollution on temporal variation, especially the use of feed containing toxic metals. The soil types and crop cultivation methods are the main factors causing toxic metal pollution on spatial variation. Although the single-factor pollution indices of all the toxic metals were within the safe limits, as per the National Soil Environmental Quality Standard (risk screening value), if the background values of soil elements in Jiaxing City are used as the standard, the pollution index of all the elements surveyed exceeds 1.0, reaching a level of mild pollution. The soil samples investigated were heavily contaminated with toxic metal compounds, and their levels increased over time. This situation poses potential ecological and health risks.

## 1. Introduction

In recent years, intensive urbanization, industrialization, and agricultural activities have had negative effects on farmland and agricultural production. In particular, contamination of soil with toxic metals in urban regions is posing a threat to the safety of agricultural products [1,2,3]. Toxic metal pollution has the characteristics of universality, concealment, surface aggregation, and irreversibility. Therefore, the pollution caused by toxic metals in farmland soil is not easy to find and solve. Based on this fact, the investigation and evaluation of toxic metal accumulation in farmland soil have been widely concerned [3].

China is an agriculturally large country with an arable area comprising 8% of the world’s total arable area and a high food production of 500 million tons per annum [4]. Jiaxing is located in the Hang-Jia-Hu plain, which is the most important agricultural production base in the Zhejiang Province of China. The farmland area in Jiaxing is less than 14% of Zhejiang Province, but it produces 30% and 48% of the total yield of vegetables and grains, respectively, in Zhejiang. Therefore, the soil quality in Jiaxing is of great significance for the safety of agricultural products and human health in Zhejiang.

In recent years, some researchers have reported the toxic metal pollution of soil and agricultural products in Jiaxing [4,5]. Liu et al. assessed the heavy metal pollution in the soil around a plastic metal factory in Jiaxing and found that the ecological risk was great [6]. Hu et al. analyzed the spatiotemporal variation and temporal changes in the sources of Cr, Pb, Cd, Hg, and As in the soil of Jiaxing based on 4359 soil samples collected in 2002 and 2012 [7]. However, the studies were local and sporadic, so the toxic metal pollution status of the soil in the entire region and the temporal and spatial variation remained unclear. The mechanism of temporal and spatial changes in toxic metal concentrations in soil still needs to be explored. The main objective of this research was to evaluate the toxic metal pollution of soil in Jiaxing and compare the local accumulation and temporal variation characteristics with the survey monitoring data of the early 1990s and the National Soil Environmental Quality Standard (GB15618-2018). The findings of the study could provide comprehensive information on the soil quality in this region and help to design strategies to minimize toxic metal transfer in the food chain.

## 2. Materials and Methods

### 2.1. Study Area

Jiaxing is located in the center of Shanghai, Hangzhou, and Suzhou, as well as other middle- to large-sized cities. It has jurisdiction over five counties (cities), including Haining, Tongxiang, Haiyan, Pinghu, and Jiashan, as well as the two districts of Nanhu and Xiuzhou, and has a total land area of 3915 km^2^.

Regarding the climate, Jiaxing belongs to the East Asian monsoon region and has four distinct seasons. Its annual average temperature is 15.9 °C, precipitation is 1168.6 mm/year, and sunshine is 2017.0 h/year. Because of the low-lying terrain, the soil in Jiaxing consists of shallow marine sediments, river alluvium, lacustrine, and paludal sediments [8]. The soil types mainly include paddy, fluvo-aquic, and coastal saline soils. Paddy soil includes silt-clayey yellow mottled, blue clayey, and silt-loamy types [7].

### 2.2. Soil Sampling and Processing

**Figure 1:**
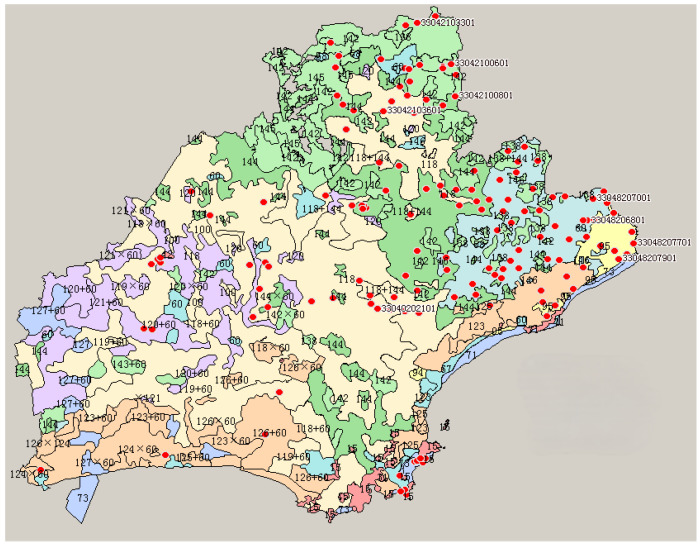
Distribution map of heavy metal elements in Jiaxing soil. The planar diagram of soil sampling locations in Jiaxing City. Red circles denote sampling points, and the numbers represent the respective point identifiers.

Sites were selected at one point per 1/15 hectare with a 1:50,000 soil map, and the Global Positioning System was used to position each sampling point. A representative plot was selected at each point, and soil samples were collected using a soil sampler in the tillage layer at a depth of 0–20 cm. Each representative sample from a point consisted of a quincunx multipoint sample (taken from at least ten sub-points) [9]. About 1 kg of each evenly mixed representative soil sample was taken for testing. In total, 250 soil samples were collected from the entire study area in the suburbs of Jiaxing (Figure 1). 

Soil samples were air-dried and screened through a 100-mesh sieve. To determine the concentration of toxic metals, the HNO_3_-HClO_4_-HF digestion method was used to prepare the samples. Available forms of various toxic metals (As, Cd, Cr, Cu, Hg, Ni, Pb, and Zn) were extracted with 0.1 mol ethylene diaminetetraacetic acid (EDTA) solution (pH 7.0) from soil–water suspensions prepared with a ratio of 1:5. The contents of As and Hg in the samples were determined through cold atomic absorption spectrophotometry (3400 ASS, Thermo Scientific, Waltham, Massachusetts, USA) [10]. The contents of Pb, Cu, Zn, Cr, Ni, and Cd were measured by the guidelines for graphite furnace atomic absorption spectrometry (240Z AA, Agilent, Palo Alto, California, USA) [11]. During all the analytical determinations, the detection limits for As, Cd, Cr, Cu, Hg, Ni, Pb, and Zn were 0.002 mg/kg, 0.01 mg/kg, 1.2 mg/kg, 0.01 mg/kg, 0.002 mg/kg, 0.3 mg/kg, 0.1 mg/kg, and 0.008 mg/kg, respectively. In addition, some national standard soil samples were also analyzed to ensure quality control. 

Soil pH was measured by potentiometry [12]. The contents of organic matter in soil samples were determined by titrimetry using sulfuric acid, potassium dichromate, and ferrous sulfate after thermal oxidation [13].

### 2.3. Data Processing and Statistical Analysis

The average values, coefficients of variation, normal distribution test, and other described statistical analyses of the toxic metal contents were performed by the SPSS 12.0 (SPSS, Chicago, IL, USA) statistical software. Variogram fitting and relevant parameters were obtained using the Statistical Analysis System (SAS 9.3, Cary, North Carolina, USA).

### 2.4. Evaluation Method

The criterion used to evaluate soil quality was the risk screening limits of the National Environmental Quality Standard for Soils. While evaluating the concentration of a single toxic metal in soil, the fuzzy synthetic evaluation method was used to determine the overall pollution level of the soil with toxic metals [14]. 

#### 2.4.1. Single-Factor Pollution Evaluation

The formula used to calculate the single-factor pollution index is as follows [15]:

Pi = Ci/Si,

where Pi is the pollution index of pollutant i, Ci is the measured concentration of the pollution index of pollutant i, and Si is the evaluation criterion of pollutant i. The Pi values for different pollution categories are as follows: Pi ≤0.7 = excellent, Pi ≤1.0 = safe, Pi ≤2 = lightly polluted, Pi ≤3 = medium-level pollution/moderately polluted, and Pi >3 = heavily polluted.

#### 2.4.2. Comprehensive Pollution Index

The single-factor index only reflects the pollution level of a single pollutant rather than the integrated pollution level of soil and crops. In contrast, the comprehensive pollution index takes into account the average and highest values of the single-factor pollution index and can highlight the role of pollutants causing serious pollution. The comprehensive pollution index is calculated as follows [16]:

P_com_ = {[(Ci/Si)^2^_max_ + (Ci/Si)^2^_ave_]/2}^1/2^,

where P_com_ is the comprehensive pollution index, Pi = Ci/Si, P_ave_ is the average value of the various pollution index Pi, and P_max_ is the maximum value among the individual pollution indices. The P_com_ values for pollution categories are as follows: P_com_ ≤ 0.7 = safe, P_com_ ≤ 1.0 = warning line, P_com_ ≤ 2.0 = lightly polluted, P_com_ ≤ 3.0 = medium-level pollution/moderately polluted, and P_com_ > 3.0 = heavy pollution/heavily polluted.

## 3. Results and Discussions

### 3.1. Temporal and Spatial Variation of Toxic Metal Pollution in Soil

#### 3.1.1. Temporal Variation of Toxic Metal Pollution in Soil

**Figure 2:**
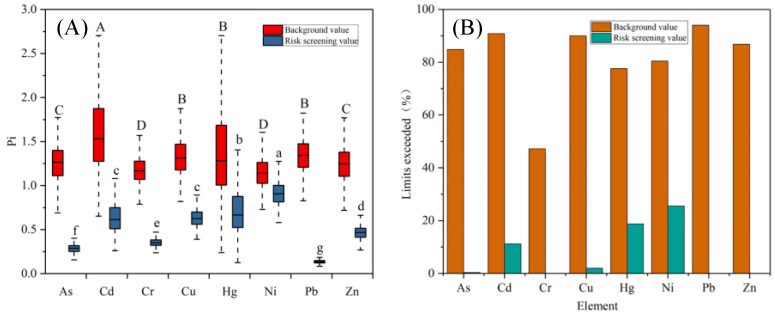
Evaluation of toxic metal element pollution in the soils of Jiaxing City. (**A**) Soil toxic metal single-factor pollution index in Jiaxing. (**B**) Excess rate of toxic metals in soil in Jiaxing City. Uppercase letters denote significance based on background values, while lowercase letters indicate significance based on risk values (*p* < 0.05).

According to Figure 2A, with the risk screening value of the National Soil Environmental Quality Standard (GB15618-2018) as the indicator, the unit pollution indices of all the toxic metals are less than 1.0. The single-factor pollution indices of all of the elements are in the safe range, in which the “excellent” category (with a pollution index less than 0.70) is up to 50%. More than 0.7 and less than 1.0 are the warning ranges. The single pollution indices of Cd, Hg, Ni, Cu, and As exceeded the warning value of 0.7 and were less than 1.0. According to Figure 2B, the proportions of Ni, Hg, Cd, Cu, and As with single pollution indices of more than 0.7 and less than 1.0 in all soil samples were 25.6%, 18.8%, 11.2%, 2%, and 0.4%, respectively. However, no sample was over-detected for Pb, Zn, or Cr.

However, according to Figure 2A, if the background values of soil elements in Jiaxing City were used as a standard, the pollution indices of all the elements investigated exceeded 1.0 and reached a mild pollution level. Among them, Cd and Hg contaminated the most seriously, reaching more than 1.5-fold of the background value. The excess rates of the metallic elements were greater than 44.6% using the local elemental background as the evaluation standard, among which Pb, CD, and Cu were over 90%. Comparing recent values of soil toxic metal tests with those obtained in Jiaxing City in 1981 (Table 1), only As and Hg levels in the soil did not increase. Cd, Zn, Cu, Pb, Ni, and Cr increased by 86.2%, 27.2%, 41.1%, 45.6%, 4.94%, and 21.1%, respectively.

**Figure 3:**
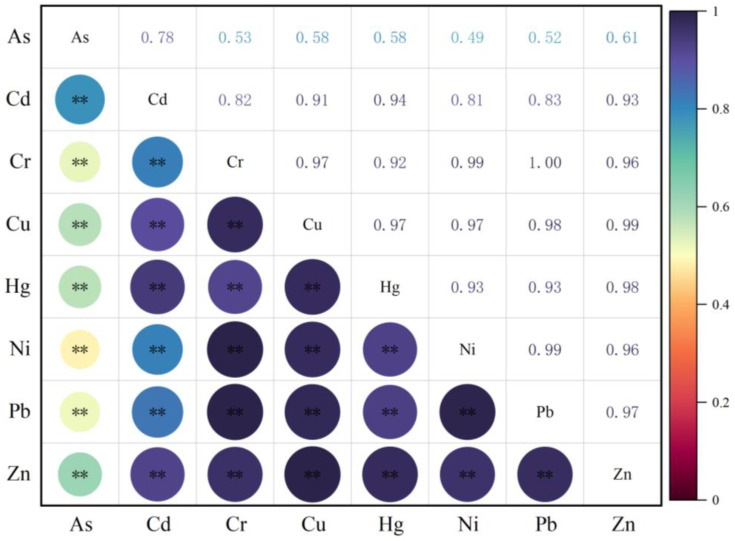
Correlation analysis of toxic metal elements in the soils of Jiaxing City. Pearson’s statistical correlation analysis was employed, where deeper colors indicate stronger correlations. (** *p* < 0.01).

Toxic metal composite contamination was severe in soils from the investigated sample sites. A total of 92.6% of the sample points had composite pollution, and the percentage of sample points with the eight toxic metals represented all over reached 16.7%. The above results demonstrated that a significant accumulation of toxic metals was occurring in the form of composite pollutants in the soils of Jiaxing City. This is also indicated by the correlation analysis in Figure 3. Cd, Cr, Cu, Ni, Pb, and Zn showed highly significant correlations with each other. This further illustrates that the above six elements are composite pollutants and that their sources share homology. The toxic metal contamination of soils is mainly composite pollution. With the single-factor pollution index (Pi) alone, it is difficult to make an objective evaluation of the overall pollution situation of soils. However, the comprehensive pollution index is able to make up for the shortage of the single-factor pollution index. With two different evaluation criteria, the calculated comprehensive pollution index was quite different. Calculations using the national secondary standard for soil environmental quality yielded a comprehensive pollution index of 0.837, indicating that the soil environmental quality in Jiaxing City is still in a safety vigilance state. However, using the background values of soil elements in Jiaxing City as the standard, the obtained comprehensive pollution index reached 1.859, indicating that the soil environment quality in Jiaxing City had reached a slightly polluted level.

#### 3.1.2. Spatial Variation of Toxic Metal Pollution

As shown in Table 2, in the soil samples from Jiaxing, the concentrations of As, Cd, and Hg varied considerably in different soil types, and the maximum values were more than 10 times higher than the minimum values. However, the contents of several other elements in different soil samples were relatively close, and the maximum value was 2–5 times the minimum value. The coefficient of variation reflects the average degree of variation between different sample points. Table 2 shows that the coefficients of variation of As, Cd, and Hg were the largest at over 0.50. The results showed large variability and spatial differentiation, indicating that outside interference considerably changed the contents of these elements in the soil. These differences might be attributed to anthropogenic activities, farming, management practices, and cropping systems [17,18].

**Figure 4:**
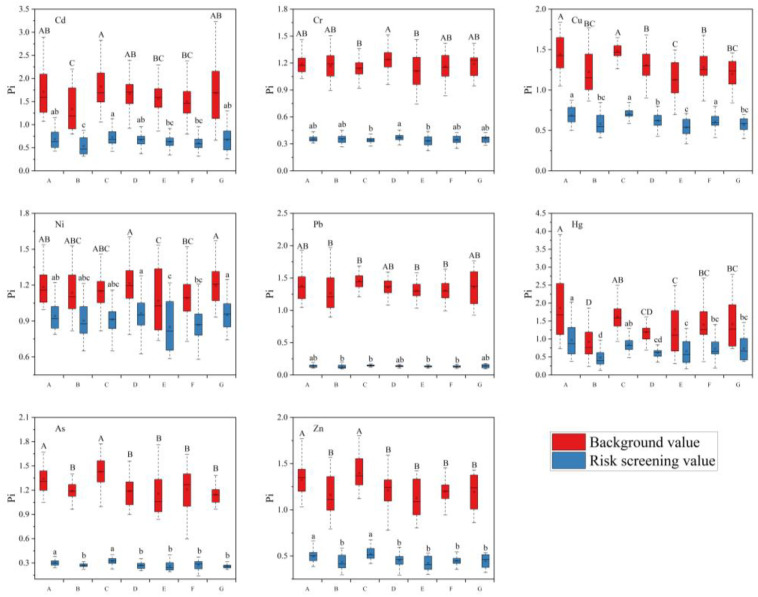
Evaluation of toxic metal element pollution in soils of different regions. (A) Nanhu District. (B) Xiuzhou District. (C) Jiashan District. (D) Haiyan District. (E) Haining District. (F) Pinghu District. (G) Tongxiang District. Uppercase letters denote significance based on background values, while lowercase letters indicate significance based on risk values (*p* < 0.05).

**Figure 5:**
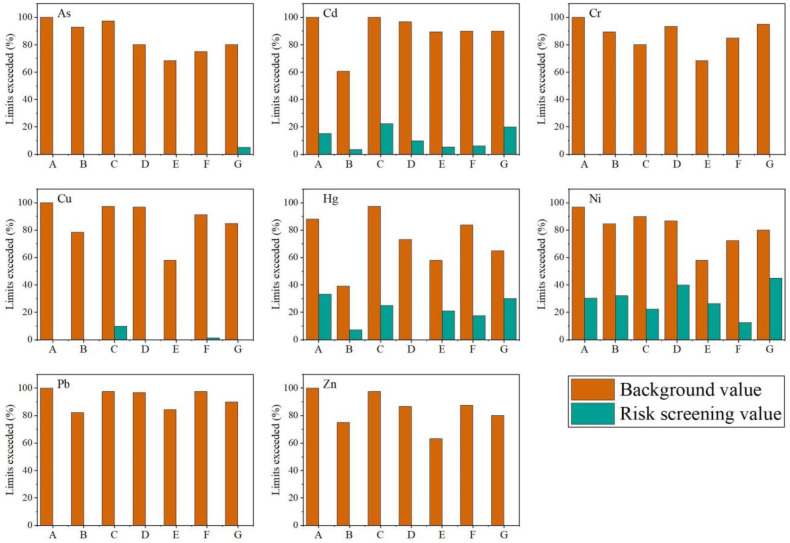
Excess rate of toxic metals in the soil in different regions. (A) Nanhu District. (B) Xiuzhou District. (C) Jiashan District. (D) Haiyan District. (E) Haining District. (F) Pinghu District. (G) Tongxiang District.

Some elements had smaller coefficients of variation (all around 0.20), suggesting that these elements were subject to consistent external influences. The spatial differentiation is low, suggesting that these elements may have the same origin in this region. As can be seen from Table 3 and Figure 4, there were some differences in the levels of toxic metals in different counties, municipalities, and districts of Jiaxing City. The main areas with higher pollution were the South Lake area and Jiashan County. The single-factor pollution indices reached 1.05–1.67 and 1.0–1.77, respectively, using the background values of toxic metals in soils from Jiaxing City as the standard. The most serious pollution of Cd, Cu, Hg, Pb, and Zn was in Jiashan County, with single-factor pollution indices of 1.06–2.82, 1.26–1.65, 0.93–2.50, 1.21–1.69, and 1.03–1.77, respectively. The pollution index of each toxic metal was relatively low in the Xiuzhou region. This trend was also reflected in the cases of exceedance rates at different localities (Figure 5). Using the background values from soils in Jiaxing City as a standard, the Cr exceedance rate in Jiashan County was 80%, while all the remaining elements exceeded 90%. The exceedances of As, Cd, Cr, Cu, Pb, and Zn in the South Lake region reached 100%, and the overall exceedances in other regions were all above 50%. The accumulation of each toxic metal element in different counties, municipalities, and regions has led to a large difference in the comprehensive pollution index in soils. The three regions with the largest comprehensive pollution index were Jiashan County, South Lake area, and Tongxiang City. Its comprehensive pollution index was calculated using the background value as the standard, and the obtained values were 2.18, 2.02, and 1.97. The obtained values were 0.92, 0.90, and 0.89, calculated using the secondary standard for soil environmental quality. The comprehensive pollution index of Xiuzhou District was the smallest, which may be related to the collection of a certain amount of pear garden soil, which was relatively small due to exogenous pollution, while the soil environmental quality remained relatively good.

### 3.2. Mechanism of Temporal and Spatial Changes in Toxic Metal Concentrations in the Soil

#### 3.2.1. Temporal Variation of Toxic Metals Affected by People’s Production

The degree of toxic metal pollution in agricultural soil is closely related to the level of regional economic development, especially the level of industry, transportation, and urbanization [19,20]. According to previous studies, first of all, industrial sources are the main reason for the accumulation of Cu, Zn, and Pb in the soil, such as the mining of minerals and the random disposal of wastes in industrial production [21]. Secondly, inappropriate agricultural measures, including sewage irrigation and fertilizer application, also become the primary causes of soil toxic metal pollution in cities with developed agriculture. The lack of water resources makes sewage an important part of irrigation water. Sewage irrigation brings a majority of harmful substances (e.g., Cd, Cr, and Zn), causing different levels of soil pollution [22]. Thirdly, vehicle exhaust emissions will significantly increase the concentration of Pb and Zn on both sides of the road [23]. Fourthly, with the development of urbanization, more household garbage will be produced. The metabolism and degradation of harmful substances are relatively slow in a closed ecosystem. Compared with the natural environment, toxic metal pollution in the urban environment is more likely to become serious [24]. In this study, with the industrialization and urbanization of Jiaxing arable land, soil toxic metal pollution has increased in the past two decades. The main pollutants are Cd, Cu, Zn, etc., which are the same as the research of Yu et al. [25]. Previous studies have reported that the main way to increase the content of toxic metals such as Cd, Cu, Hg, and Zn in agricultural soils in Zhejiang Province is through crop harvesting and leaching, accounting for 74.43–83.62% of the total output [26]. Cd is the priority control pollutant in agricultural soil in this study, which is the same as that obtained by Kim et al. [27]. They found that Cd pollution from industrial activities is serious or extremely serious to agricultural soil.

According to the survey, before 1990, pigs and poultry were fed coarse grains and green feed without any commercial feed, so there was no chance of toxic metal pollution. After 1990, the number of large-scale pig farms in South Lake, Jiashan, and Tongxiang increased gradually. A small amount of toxic metals can be added to the feed, which can promote the growth and development of pigs. Therefore, the fermentation of pig manure containing toxic metals as organic waste materials directly applied to agricultural fields resulted in a significant increase of toxic metals in agricultural soils. According to Wang et al. [28], the organic fertilizer samples contained up to 364–2213 mg·kg^−1^ Cu and 319–4359 mg·kg^−1^ Zn. Through the above analysis, it can be inferred that As pollution is mainly concentrated in these areas, which may be due to the long-term high-intensity use of pig manure, resulting in the serious accumulation of toxic metals in these areas.

#### 3.2.2. Spatial Variation of Toxic Metals Affected by Soil Types and Crop Cultivation Methods

Data obtained from 250 samples were subjected to statistical analysis using the risk screening value of the National Soil Environmental Quality Standard as the limit to calculate the arithmetic mean, single-factor pollution index (Pi), and comprehensive pollution index (Pcom) of the samples, as shown in Table 3. The soil types ranged from blue–purple clay distributed in the lake group, disc edge, and low ground in the mainland, and silt-clayey yellow mottled, blue clayey, and green powder paddy soils on the central water network plains to the semi-sandy soil and silt-loamy paddy soil on the coastal (along with the river plains); the comprehensive pollution indices obtained for these soils were 0.614, 0.697, 0.602, 0.584, 0.626, 0.632, 0.464, and 0.495, respectively. Toxic metal pollution shows a clear spatial variation, decreasing from the mainland to the coastal plain. In addition, research has demonstrated that transportation plays a significant role as a conduit for heavy metals to move from land to rivers, closely correlating with distance, freight volume, and traffic emissions [29]. Soil texture also transitions from sticky clay loam soil to light loam soil (except for some silt-loamy paddy soil with a light texture). The gradual decline of the comprehensive pollution index (i.e., the decrease of the degree of soil pollution) is, therefore, related to the type and texture of the soil as well as the terrain (water flow). More toxic metals may accumulate in the soil on low-lying land. 

Table 3 reflects the content status and quality evaluation of each element in different soil types. It can be seen that the content of toxic elements and the rate of exceeding the standard in blue clayey paddy soil are relatively high. Taking the background value of soil and the second level limit of soil environmental quality as the standard calculation, the comprehensive pollution indices obtained reached 1.78 and 0.78, respectively. Secondly, the relatively heavy pollution is green powder paddy soil and yellow sand moisture soil, while the element content of silt-loamy paddy soil and semi-sandy soil is close to the background value, basically no pollution. The main reason for this phenomenon may be that the pollutant-carrying capacity of different terrain parts of different soils is different; it may also be caused by the difference in element content in the parent material developed by each soil itself, or it may be related to the physical and chemical properties of the soil. For example, the blue clayey paddy soil is sticky, and the amount of organic matter and cation exchange is the highest of all the soils surveyed, which is conducive to its adsorption and fixation of more foreign elements. While silt-loamy paddy soil and semi-sandy soil are the most sandy of all soils, with relatively low organic matter content, the ability to adsorb and fix foreign elements is very weak, and foreign elements are not easy to accumulate in the soil.

**Figure 6:**
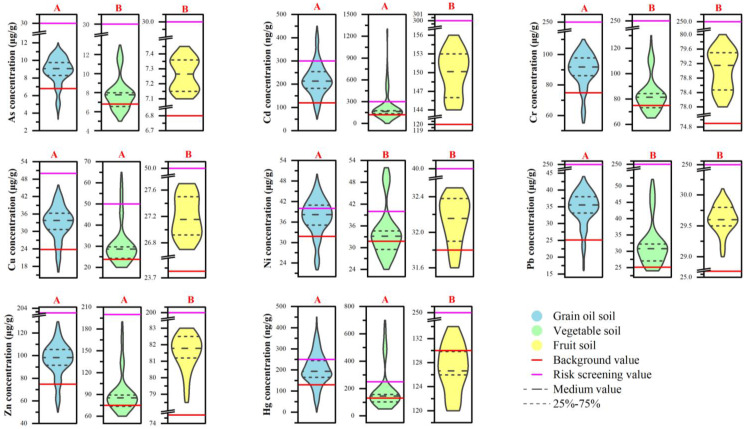
Toxic metal content of soils with different utilization. Red uppercase letters indicate significant differences in heavy metal concentrations among different soil types under various land uses (*p* < 0.05).

The effects of different crop cultivation methods on the contents of different toxic metals in soils were significantly different. It can be seen from Figure 6 that the content in the soils increased significantly compared to the background values 30 years ago, regardless of the crop cultivation method. Overall, As, Cd, Cr, Cu, Hg, Ni, Pb, and Zn were most abundant in soils grown from grain and oil crops. Toxic metals are least abundant in fruit tree-grown soils. However, taking the screening values in soil environmental quality as the standard, the exceedances of Cd, Cu, Ni, and Hg in vegetable-grown soils were significantly higher than those in soils grown in grain and oil crops and fruit trees.

## 4. Conclusions

This study investigated the temporal and spatial variations and mechanisms of toxic metal (As, Cd, Cr, Cu, Hg, Ni, Pb, and Zn) contamination in 250 agricultural soil samples from Jiaxing City. While the levels of all eight toxic metals were found to be within safe limits compared to national risk screening values, there has been a gradual deterioration in toxic metal pollution in the investigated area since the early 1990s, accumulating significantly in a composite pollution manner. Particularly alarming was the contamination of Cd and Hg, which exceeded background levels by more than 1.5 times, potentially elevating environmental and health risks. Moreover, Jiashan County, Nanhu District, and Tongxiang City exhibited the most severe comprehensive pollution, with pollution indices reaching 2.18, 2.02, and 1.97, respectively. Industrialization and urbanization were identified as the primary drivers of temporal variations in toxic metal pollution, while soil types and crop cultivation methods were found to dominate spatial variations in toxic metal pollution. Future research should prioritize investigating the pollution characteristics of toxic metals in crops, thereby enabling the formulation of more effective governance and remediation strategies.

## Figures and Tables

**Table 1:** Comparison of Soil Environmental Quality Evaluation Standards.

Item	As	Cd	Cr	Cu	Hg	Ni	Pb	Zn
Risk screening value (µg/g)	30	0.300	250	50	0.250	40	250	200
Background value (µg/g) *	6.8	0.120	74.9	23.8	0.130	31.8	25.1	74.8
Soil test values in 1981 (µg/g) **	9.87	0.116	72.34	22.67	0.195	34.59	23.08	74.11

µg/g is the quality of toxic metals in the tested soil. “*” is the background value of soil toxic metal concentration in Hangjiahu plain in the early 1990s; “**” is the background content of toxic metals obtained by the Jiaxing environmental monitoring station after sampling 28 points in Jiaxing, Jiashan, Pinghu, Haiyan, Haining, and Tongxiang counties (cities) in 1981.

**Table 2:** Descriptive statistical analysis of soil total toxic metals in Jiaxing City (µg/g).

Elements	Mean	STD	Max.	Min.	Variation Coefficient	Skewness	Kurtosis
As	8.76	4.63	77.33	4.07	0.53	13.11	194.63
Cd	0.22	0.16	1.26	0.078	0.53	4.29	30.08
Cr	87.56	11.75	125.59	55.73	0.13	−0.061	0.19
Cu	32.03	6.82	61.72	16.58	0.21	0.94	2.49
Hg	0.19	0.098	0.65	0.031	0.51	1.84	4.87
Ni	36.31	5.77	51.01	23.20	0.16	0.11	−0.18
Pb	33.62	5.48	50.76	16.61	0.16	0.053	0.51
Zn	94.28	18.50	188.21	53.80	0.19	1.22	4.27

**Table 3:** Statistical analysis of soil toxic metal elements in counties (µg/g).

Element		Nanhu	Xiuzhou	Jiashan	Haiyan	Haining	Pinghu	Tongxiang
As	Content range	7.2–12.5	4.6–10.7	6.8–12.1	6.1–10.6	5.7–12.0	4.1–11.2	5.1–77.3
	Mean	9.2	8.1	9.7	8.1	7.9	8.2	11.7
Cd	Content range	0.1–0.7	0.1–0.4	0.1–1.3	0.1–0.5	0.1–0.5	0.1–0.8	0.1–0.4
	Mean	0.2	0.2	0.3	0.2	0.2	0.2	0.2
Cr	Content range	76.8–109.3	66.8–112.7	57.1–125.6	59.1–117.6	55.7–109.6	62.5–106.5	70.6–106.3
	Mean	89.2	87.4	84.3	92.7	83.0	87.0	89.3
Cu	Content range	25.0–43.8	20.5–42.1	22.4–61.7	21.3–39.9	16.5–35.5	16.8–53.3	20–42.3
	Mean	34.4	29.1	37.9	31.1	26.9	30.9	29.3
Hg	Content range	0.1–0.5	0.1–0.5	0.1–0.7	0.1–0.2	0.1–0.3	0.1–0.5	0.1–0.5
	Mean	0.2	0.1	0.3	0.2	0.2	0.2	0.2
Ni	Content range	31.6–48.9	26.0–48.5	26.0–46.4	25.0–51.0	23.4–48.7	23.2–48.3	29.7–49.9
	Mean	37.7	36.1	36.5	38.6	34.0	34.7	38.0
Pb	Content range	26.2–48.3	22.4–49.4	22.0–42.3	22.9–39.9	16.6–39.7	17.6–41.1	23.2–44.2
	Mean	34.5	31.7	36.2	33.7	30.7	32.6	34.0
Zn	Content range	77.1–132.4	59.2–117.4	62.1–134.9	58.2–118.9	60.2–106.2	53.8–188.2	64.3–162.1
	Mean	99.1	87.0	103.5	90.6	84.4	92.0	93.5

The number of soil samples from Nanhu, Xiuzhou, Jiashan, Haiyan, Haining, Pinghu, and Tongxiang were 33, 28, 19, 20, 30, 80, and 40, respectively.

## Data Availability

The data used to support the findings of this study are included within the article. Some or all the data or models that support the findings of this study are available from the corresponding author upon reasonable request.
